# Effects of Dietary Ochratoxin A on Growth Performance and Intestinal Apical Junctional Complex of Juvenile Grass Carp (*Ctenopharyngodon idella*)

**DOI:** 10.3390/toxins13010011

**Published:** 2020-12-24

**Authors:** Xin Liu, Pei Wu, Wei-Dan Jiang, Yang Liu, Jun Jiang, Sheng-Yao Kuang, Ling Tang, Xiao-Qiu Zhou, Lin Feng

**Affiliations:** 1 Animal Nutrition Institute, Sichuan Agricultural University, Chengdu 611130, China; xinliu6966@foxmail.com (X.L.); wupei0911@sicau.edu.cn (P.W.); WDJiang@sicau.edu.cn (W.-D.J.); yangliu20000@126.com (Y.L.); jjun@sicau.edu.cn (J.J.); 2 Fish Nutrition and Safety Production University Key Laboratory of Sichuan Province, Sichuan Agricultural University, Chengdu 611130, China; 3 Key Laboratory of Animal Disease-Resistant Nutrition, Ministry of Education, Chengdu 611130, China; 4 Key Laboratory of Animal Disease-Resistant Nutrition and Feed, Ministry of Agriculture and Rural Affairs, Chengdu 611130, China; 5 Animal Nutrition Institute, Sichuan Academy of Animal Science, Sichuan Animtech Feed. Co., Ltd., Chengdu 610066, China; shengyao.kuang@gmail.com (S.-Y.K.); lingtang.fish@gmail.com (L.T.); 6 Key Laboratory of Animal Disease-resistance Nutrition, Chengdu 611130, China

**Keywords:** ochratoxin A, intestine, apical junctional complex, tight junction, adhesive junction, juvenile grass carp (*Ctenopharyngodon idella*)

## Abstract

Ochratoxin A (OTA) contamination widely occurs in various feed ingredients and food crops, potentially posing a serious health threat to animals. In this research, 1260 juvenile grass carp were separately fed with seven distinct experimental diets (0, 406, 795, 1209, 1612, 2003 and 2406 µg of OTA/kg of diet) for 60 consecutive days to evaluate OTA’s toxic effect on the intestinal apical junctional complex (including the tight junction (TJ) and the adherents junction (AJ)) and the underlying action mechanisms. Our experiment firstly confirmed that OTA caused fish growth retardation and disrupted the intestinal structural integrity. The detailed results show that OTA (1) depressed the feed efficiency, percentage weight gain and specific growth rate; (2) accumulated in the intestine; (3) caused oxidative damage and increased intestinal permeability; and (4) induced the RhoA/ROCK signaling pathway, destroying intestinal apical junctional complexes. Notably, OTA intervention did not result in changes in the gene expression of claudin-3c (in the proximal intestine (PI)), claudin-b and ZO-2b (in the mid intestine (MI) and distal intestine (DI)) in the fish intestine.

## 1. Introduction

Mycotoxins, produced by fungi, are secondary harmful products that are the most poisonous biological toxins in food pollution. Various toxicological and pathological effects can occur after animals ingest food or feed contaminated with toxins [1,2]. Mycotoxin pollution seriously threatens animal growth and health [3,4]. As plant protein sources are gradually used to replace animal-derived proteins in aquafeed, mycotoxins are receiving more prominent attention in aquaculture [5]. Ochratoxin A (OTA) is a mycotoxin produced by toxin-producing strains (such as Aspergillus and Penicillium) that is widely present in various feed ingredients and food crops [6]. The prevalence of OTA in feed, corn and wheat has been reported to reach up to 1582, 889 and 364 µg/kg, respectively [7]. Numerous studies regarding the toxic effects of OTA in mammals have shown that OTA can cause diarrhea, bleeding, immunosuppression and growth retardation [8,9]. To date, the number of studies that have focused on the toxic impacts of OTA on aquatic animals has been low. Limited efforts have ascertained that OTA could cause histopathological damage to the kidney [10] and reduce the growth performance of channel fish (*Ictalurus punctatus)* [11]. Reportedly, fish growth acceleration has a highly coordinated link with intestinal health [12,13], and the intestine is the first target organ for foodborne toxic substances [14]. Studies have found that OTA can destroy the apparent intestinal morphology of animals [15,16,17]. Accordingly, we speculate that OTA may destroy the intestinal physical barrier function of the fish intestine.

The harm to the intestinal physical barrier is manifested by increased intestinal mucosal permeability, which can be well reflected by the increase in D-lactate content and DAO activity in the serum [18]. Accumulating evidence has demonstrated that the function of the intestinal physical barrier is maintained by the intestinal apical junctional complex (AJC, composed of the tight junction (TJ) and the adhesive junction (AJ)), which plays a vital regulatory role in maintaining the physical barrier function [19]. The TJ is located at the top of the intestinal epithelium and is responsible for the permeability of the intestinal mucosal barrier [20]. The TJ mainly includes the zonula occludens (ZOs) and three transmembrane proteins (occludin, junctional adhesion molecules (JAM) and claudins) [21,22]. The AJ is below the TJ and is mainly composed of cadherin (E-cadherin, N-cadherin, etc.) [23] and linker proteins (α-catenin, β-catenin, etc.) [24]. The contraction of actomyosin in mammalian enterocytes controls AJC integrity, via the activation of MLCK and NMII by the RhoA/ROCK signaling pathway [19,25]. Meanwhile, this pathway is activated depending on the GTP-RhoA level [26]. However, it is still unclear whether the destruction by OTA of fish intestinal physical barrier function has relations to the AJC and related signaling. It has been reported that OTA can reduce the protein levels of ZO-1 and occludin in human Caco-2 cells [27,28] and porcine intestinal epithelial cells (IPEC-J2) [29], and decrease the protein levels of β-catenin and E-cadherin in Madin-Darby canine kidney cells (MDCK) [30]. However, those studies are still lacking in systematicity and did not examine the involved mechanisms. Thence, it is necessary to systematically study the causal relationship between OTA and intestinal physical barrier function, and conduct a thorough investigation researching the molecular mechanisms in animals.

Hence, the purpose of this research was first to elaborate the toxicological effect of OTA on fish intestinal morphology, permeability, the AJC and the involved signaling. In addition, we also evaluated the maximum controlled level of OTA in aquafeed, which could provide part of the foundation for healthy grass carp breeding.

## 2. Results

### 2.1. Growth Performance and OTA Intestinal Residue

As shown in Table 1, there was no significant difference between the initial body weight (IBW) of each treatment. Relative to the control group, when the level of ochratoxin A increased to 1209 µg/kg of diet, the feed efficiency, percent weight gain, final body weight and specific growth rate decreased (*p* < 0.05). When the level of ochratoxin A increased to 1612 µg/kg of diet, the feed intake (FI), intestinal length index (ILI) and intestinal weight (IW) decreased (*p* < 0.05). Moreover, the residual amount of ochratoxin A in three intestinal segments increased continuously with an increase in the ochratoxin A level in the diet. It is noteworthy that when the dose was lower than 406 µg/kg of diet, its residue was not detected in the intestine.

### 2.2. Serum D-Lactate Concentration and Diamine Oxidase (DAO) Activity

**Figure 1:**
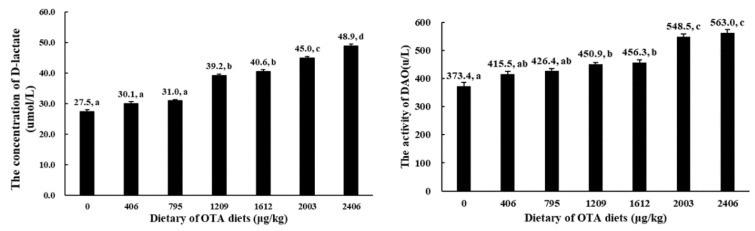
Effects of OTA (µg/kg of diet) on intestinal mucosal permeability. Data represent means (*n* = 6); error bars indicate S.D. Values above bars with different letters are significantly different (*p* < 0.05).

The D-lactate concentration and DAO activity in the serum are presented in Figure 1. The D-lactate concentration and DAO activity in the serum of the fish were decreased with levels of OTA up to 1209 µg of OTA/kg of diet.

### 2.3. Dietary OTA-Induced Intestinal Hyperemia and Histopathological Changes

**Figure 2:**
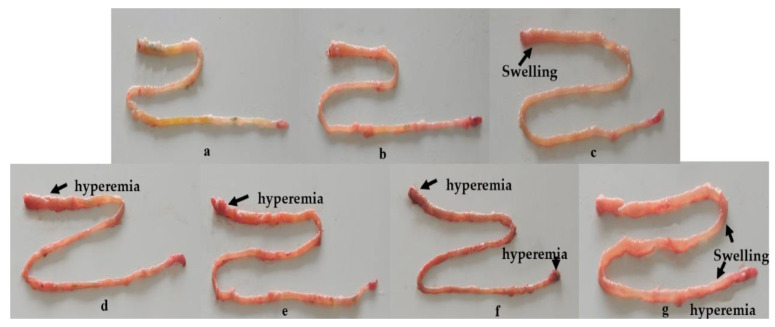
The intestinal lesion (hyperemia and swelling) symptoms of juvenile grass carp (*Ctenopharyngodon idella*) fed diets containing graded levels of OTA for 60 days. (**a**) Control; (**b**) 406 µg/kg of diet; (**c**) 795 µg/kg of diet; (**d**) 1209 µg/kg of diet; (**e**) 1612 µg/kg of diet; (**f**) 2003 µg/kg of diet; (**g**) 2406 µg/kg of diet.

**Figure 3:**
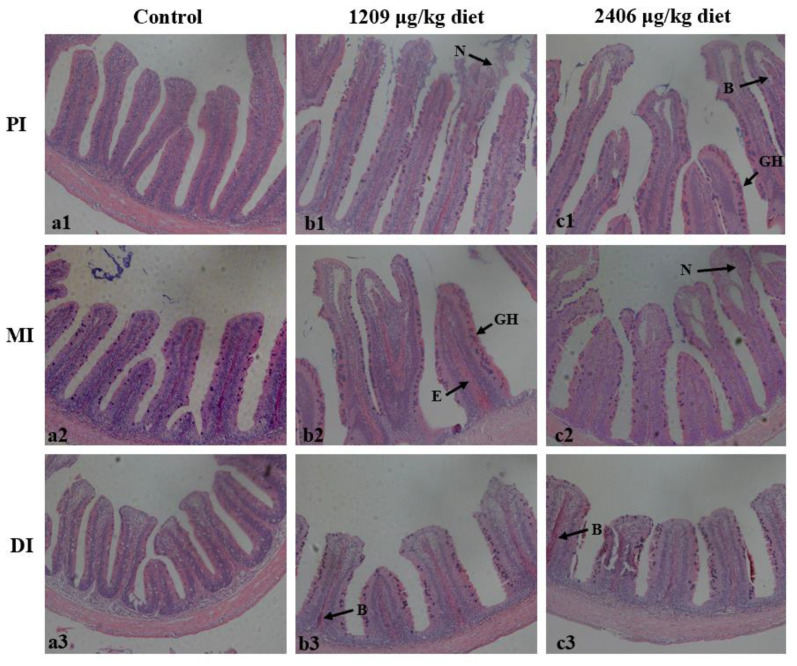
The histology analysis of the PI, MI and DI in juvenile grass carp fed diets containing graded levels of OTA (µg/kg diet) for 60 days. Control (**a1**–**a3**), 406 µg/kg of diet (**b1**–**b3**); 2406 µg/kg (**c1**–**c3**) of diet. In each panel, N: necrosis; GH: goblet cell hyperplasia; B: blood capillary hyperemia; and E: edema in the lamina propria. H&E staining; magnification × 200.

Compared with controls, the intestine showed obvious swelling in the 795 µg of OTA/kg of diet group, hyperemia with 1209 and 1612 µg of OTA/kg of diet, and hyperemia and swelling with 2003 and 2406 µg of OTA/kg of diet (Figure 2). Upon further histopathological observation (Figure 3), it was found that when the dose was lower than 1209 µg of OTA/kg of diet, histopathological changes were not observed. We observed the following phenomena: with doses of up to 1209 µg of OTA/kg, necrosis (N) in the proximal intestine (PI), goblet cell hyperplasia (GH) and edema in the lamina propria (E) in the mid intestine (MI), and blood capillary hyperemia (B) in the distal intestine (DI) could be found. When the treatment groups were administered a dose of 2406 µg of OTA/kg, blood capillary hyperemia (B) and goblet cell hyperplasia (GH) in the PI, necrosis (N) in the MI, and blood capillary hyperemia (B) in the DI could be observed.

### 2.4. Antioxidant-Related Parameters

As demonstrated in Table 2, the reactive oxygen species (ROS), protein carbonyl (PC) and malondialdehyde (MDA) contents in all the intestinal segments increased with increasing OTA levels. Noteworthily, compared with controls, these indicators were markedly increased when increasing the diet OTA levels to 1209 µg/kg (*p* < 0.05).

### 2.5. Relative mRNA Levels of AJC-Related Parameters in the Intestine

#### 2.5.1. OTA Decreased the mRNA Expression Levels of Tight Junctions

**Figure 4:**
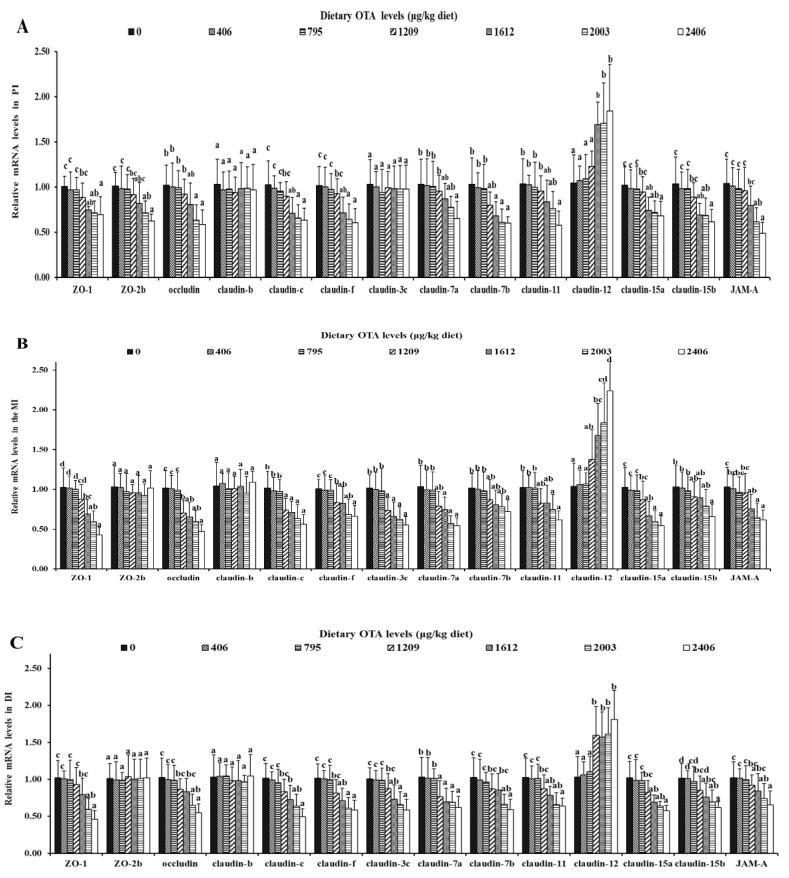
Relative mRNA levels of tight junction (TJ)-related proteins in PI (**A**), MI (**B**) and DI (**C**) of juvenile grass carp fed diets containing graded levels of ochratoxin A. Data represent means of six fish in each group; error bars indicate S.D. Values above bars with different letters represent statistically significant differences (*p* < 0.05).

As presented in Figure 4, compared with the control group, the mRNA expression levels of ZO-1, claudin-c, claudin-f, claudin-7b, claudin-11, claudin-15a, claudin-15b and JAM-A in the PI were remarkably down-regulated (*p* < 0.05). When the diet level increased to 1612 µg of OTA/kg of diet, the gene expression levels of claudin-12 were significantly up-regulated. In addition, when the diet levels increased to 2003, 2406 and 2406 µg/kg of diet, the gene expression levels of claudin-7a, occluding and claudin-11 in the PI were remarkably down-regulated (*p* < 0.05). In the MI, when the level of ochratoxin A in the diet increased to 1209 µg/kg of diet, the mRNA levels of claudin-c, claudin-f, claudin-3c and occludin were significantly down-regulated (*p* < 0.05), and the gene expression level of claudin-12 was significantly up-regulated (*p* < 0.05). When the concentration was increased up to 1612 µg of OTA/kg of diet, the mRNA levels of ZO-1, claudin-7a, claudin-15a and JAM-A markedly declined (*p* < 0.05). When the levels of ochratoxin A in the diet increased to 2406, 2003 and 2406 µg/kg of diet, the mRNA expression levels of claudin-7b, -11 and -15b were significantly reduced (*p* < 0.05), respectively. When the amount of ochratoxin A in the diet was increased to 1612 µg/kg of diet, ZO-1, claudin-c, claudin-f, claudin-3c, claudin-7a, claudin-11, claudin-15a and claudin-15b mRNA levels were substantially down-regulated in the DI (*p* < 0.05), and claudin-12 gene expression was significantly up-regulated (*p* < 0.05). When the levels of ochratoxin A in the diet increased to 2406, 2003 and 2003 µg/kg of diet, the gene expression levels of occludin, claudin-7b and JAM-A were dramatically lowered (*p* < 0.05), respectively. It was also noted that OTA had no effect on the gene expression of claudin-b, claudin-3c (PI) and ZO-2b (in the MI and DI) (*p* > 0.05).

#### 2.5.2. OTA Decreased the mRNA Expression Levels of Adhesive Junctions

**Figure 5:**
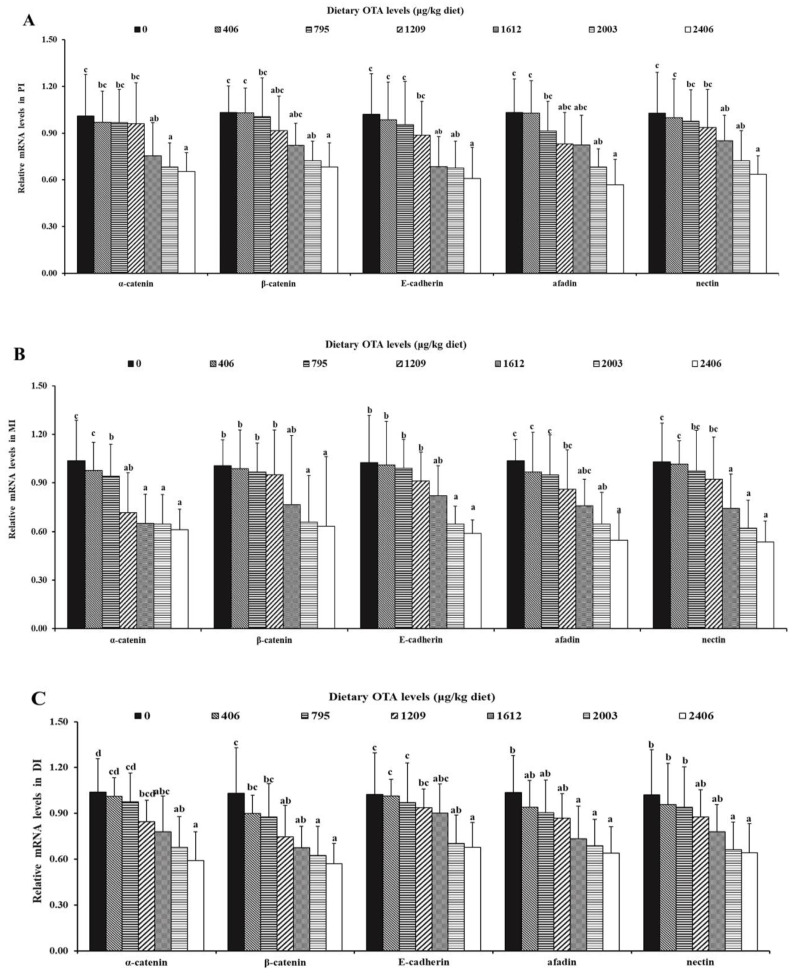
Relative mRNA levels of AJ-related proteins α-catenin, β-catenin, E-cadherin, afadin and nectin in PI (**A**), MI (**B**) and DI (**C**) of juvenile grass carp fed diets containing different levels of ochratoxin A. Data represent means of six fish in each group; error bars indicate S.D. Values above bars with different letters represent statistically significant differences (*p* < 0.05).

As shown in Figure 5, in the PI, the mRNA levels of α-catenin, β-catenin, E-cadherin, afadin and nectin were markedly decreased when elevating the dietary OTA levels up to 1612, 2003, 1612, 2003 and 1612 µg/kg of diet (*p* < 0.05), respectively. Similarly, in the MI, the gene expression levels of α-catenin, β-catenin, E-cadherin, afadin and nectin were markedly decreased when the dietary OTA levels reached 795, 2003, 2003, 2003 and 1612 µg/kg of diet (*p* < 0.05), respectively. Besides, the gene expression levels of α-catenin, β-catenin, E-cadherin, afadin and nectin in the DI were markedly decreased as the ramping dietary OTA levels reached up to 1612, 1209, 2003, 1612 and 2003 µg/kg of diet (*p* < 0.05), respectively.

#### 2.5.3. OTA Increased the mRNA Expression Levels of AJC and Relevant Signaling Molecules

**Figure 6:**
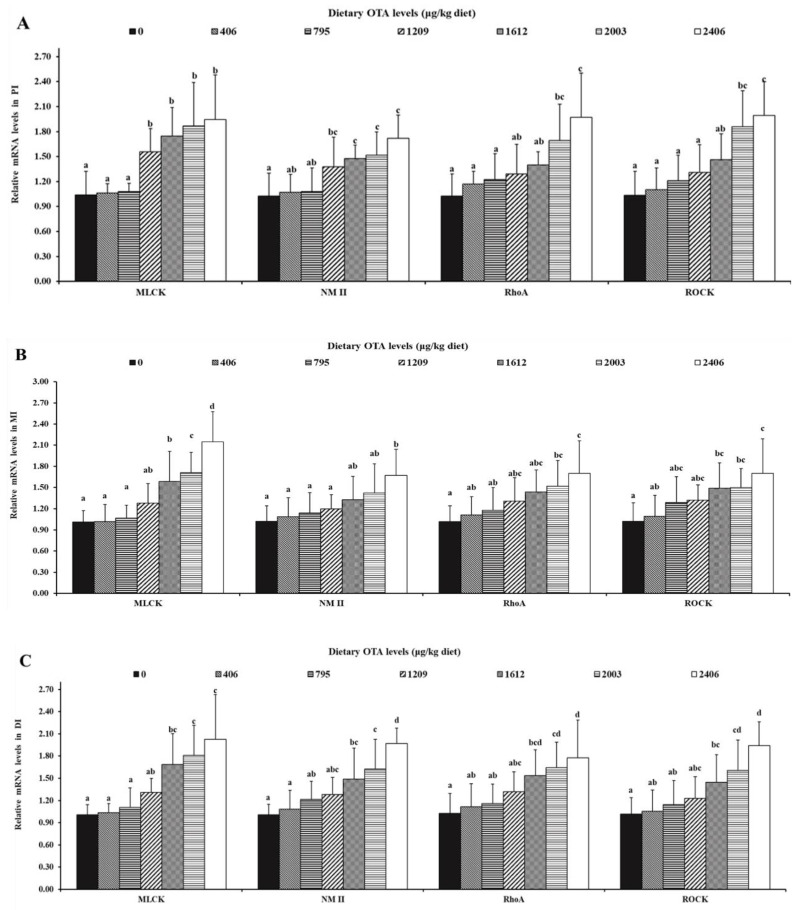
Relative mRNA levels of AJC-related signaling molecules in PI (**A**), MI (**B**) and DI (**C**) of juvenile grass carp fed diets supplemented with different levels of OTA. Data represent means of six fish in each group, error bars indicate S.D. Values above bars with different letters represent statistically significant differences (*p* < 0.05).

As is presented in Figure 6, in the PI, the gene expression levels of MLCK, NMII, RhoA and ROCK were markedly decreased when increasing the dietary OTA concentrations up to 1209, 1209, 1209 and 1612 µg/kg of diet (*p* < 0.05), respectively. In the MI, the gene expression levels of MLCK, NMII, RhoA and ROCK were markedly decreased as the dietary OTA concentrations reached 1612, 2406, 2003, 2003 and 1612 μg/kg of diet (*p* < 0.05), respectively. Besides, in the DI, the gene expression levels of MLCK, NMII, RhoA and ROCK markedly decreased as the increasing dietary OTA levels reached up to 1612 μg/kg of diet (*p* < 0.05).

### 2.6. OTA Decreased the Protein Levels of GTP-RhoA

**Figure 7:**
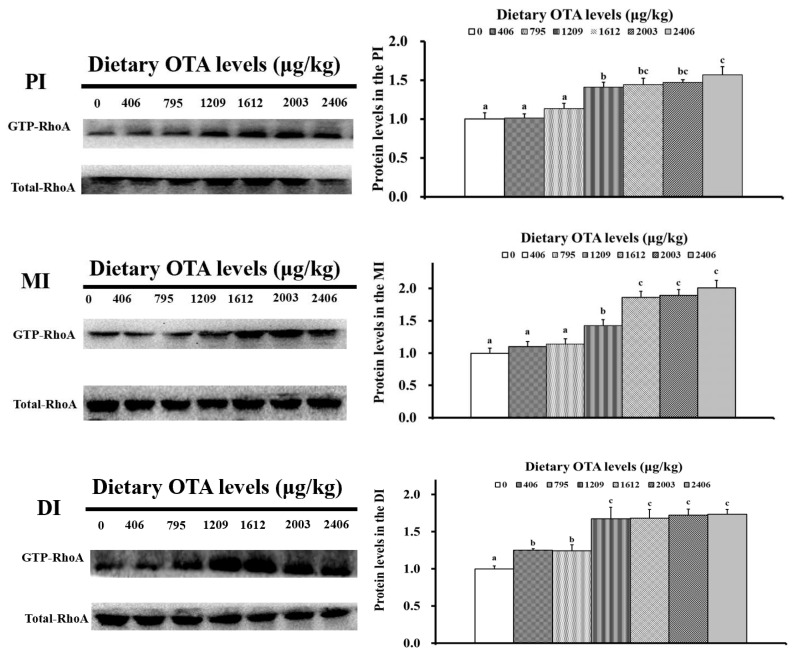
Western blot analysis of GTP-RhoA in the PI, MI and DI of juvenile grass carp (*Ctenopharyngodon idella*) fed diets supplemented with different levels of OTA for 60 days. Values above bars with different letters represent statistically significant differences (*p* < 0.05).

As presented in Figure 7, the protein levels of GTP-RhoA in all the intestinal segments were decreased with rising dietary OTA concentrations. The GTP-RhoA protein levels all substantially decreased with elevated dietary OTA concentrations up to 1612 µg/kg of diet (*p* < 0.05).

### 2.7. Correlation Analyses

As shown in Table S1, the mRNA levels of the TJs (except claudin-b, claudin-3c (PI) and ZO-2b (in the MI and DI)) and AJs were negatively correlated with RhoA, ROCK, NMII and MLCK mRNA levels and GTP-RhoA protein levels. By contrast, pore-forming TJs (not including claudin-15a and -15b) were positively correlated with RhoA, ROCK, MLCK and NMII gene expression levels and GTP-RhoA protein levels.

## 3. Discussion

### 3.1. OTA Caused Poor Growth Performance in Fish

Research has shown that toxic substances can induce fish growth inhibition [31]. In our study, OTA levels up to 1209 µg/kg of diet inhibited fish growth performance (indicated by a decreased feed efficiency (FE), percent weight gain (PWG) and feed intake (FI)) and intestinal growth development (decreased the intestinal length (IL), IW, ILI and intestinal somatic index (ISI)). Based on the PWG, the maximum controlled dose of OTA in juvenile grass carp (25–125 g) was evaluated to be 808.56 µg/kg of diet, and when the OTA dose was not higher than 808.56 µg/kg, it did not affect fish growth.

A study found that grass carp growth is related to intestinal structural integrity [32]. The destruction of the intestinal structure may be related to histopathological damage [33]. Therefore, we next investigated the effect of OTA on it.

### 3.2. OTA Led to Fish Intestine Histopathological Lesions

The accumulation of harmful substances may cause intestinal histopathological damage [34]. This study found that OTA began to accumulate in the fish DI when the OTA content in the diet was ≥795 µg/kg, and began to accumulate in the fish PI and MI when the content was ≥1209 µg/kg. At the same time, it was observed that OTA could cause significant swelling and hyperemia of the intestines in fish. Pathological occurrences in fish intestines may be induced by toxic substances [35]. Further histological examination showed that diets with OTA contents ≥ 1209 µg/kg induced pathological changes, such as epithelial cell necrosis, lamina propria edema, and goblet cell hyperplasia [31]. Meanwhile, recent research recorded that histological lesions (enlarged melano-macrophage centers concentrated in the interstitium) could be induced by OTA in the kidneys of rainbow trout [36].

Histopathological damage is always tightly related to oxidative damage by toxins in rats [37]. It is widely known that oxidative damage (indicated by MDA and PC) can be caused by excess ROS [38]. In our research, OTA doses up to 1209 µg/kg markedly increased the contents of ROS, MDA and PC in the grass carp intestines, showing that OTA induced the oxidative damage of the fish intestines. Oxidative damage leads to the barrier disruption of the fish intestine [39]. A complete intestinal mucosa is responsible for the normal function operations of the intestine [40]. Epithelial permeability increased after the impairment of the intestinal barrier in weaned piglets [41]. Plasma DAO activity and D-lactic acid content are often used as indicators to judge intestinal permeability in piglets [42]. When the mucosa is impaired, DAO activity and the D-lactic acid concentration will increase in the serum and decrease in the mucosa. The study found that a dietary OTA content of 1209 µg/kg significantly increased DAO activity and the D-lactate concentration in the serum, suggesting that OTA might cause damage to the intestinal structure and increase permeability in fish. As stated above, the physical barrier function of the intestine is closely correlated with the AJC and RhoA/ROCK pathway. Therefore, we next inspected the influence of OTA on the intestinal AJC and explored the potential mechanisms in fish.

### 3.3. OTA Disrupted Fish Intestine AJC, Partly via RhoA/ROCK Pathway

Intestinal epithelial cells are interconnected by an apical junction complex (AJC) that includes tight junctions (TJs) and adherent junctions (AJs) in animals [43]. The disruption of the AJC can destroy the integrity of the intestinal structure of animals [44]. Previous studies in fish have shown that the destruction of the intestinal tight junction complex might be related to the down-regulation of the barrier-forming TJ-related proteins occludin and ZOs and up-regulation of the pore-forming TJ-related protein claudin-12 [45]. This experiment found that when the OTA content reached 1209 µg/kg, ZO-1, occludin, claudin-7a, claudin-7b, claudin-c, JAM, claudin-11, claudin-15a, claudin-15b and claudin-f gene expression was down-regulated, and claudin-12 gene expression was up-regulated. We also observed that OTA had an impact on the gene expression of the AJ-related proteins α-catenin, β-catenin, E-cadherin and afadin, which indicated that OTA disrupted the AJC structure of grass carp.

Studies have shown that in mammalian intestinal epithelial cells, the activation of RhoA/ROCK signaling leads to the activation of MLCK [19,25]. Our research on AJC-related signaling molecules showed that with an increase in OTA dose, the mRNA and protein levels of RhoA as well as the gene expression of the ROCK, MLCK and NMII signaling molecules in the three intestinal segments were observably increased. Correlation analysis showed that the gene expression of barrier-forming TJ proteins (except claudin-b, claudin-3c (PI) and ZO-2b (in the MI and DI)) and all the studied AJs was negatively related to GTP-RhoA protein levels, and pore-forming TJ proteins’ (except claudin-15a and -15b) gene expressions were positively related to GTP-RhoA protein levels in the three fish intestinal sections. Consequently, the destruction of the AJC in the intestine by OTA might occur through the RhoA/ROCK signaling pathway.

Intriguingly, we found some diversity in the effects of OTA on gene expression. Firstly, OTA had no influence on claudin-b expression in the intestines, which might be ascribed to cortisol. Research observed that OTA promoted rat cortisol secretion [46], which affected claudin-b expression levels in goldfish (*Carassius auratus*) [47]. Therefore, the lack of change in claudin-b induced by OTA be related to cortisol, but the specific mechanism of action needs further research. Secondly, OTA only inhibited claudin-3c gene expression in the fish MI and DI, which might be related to the role of glucocorticoids. Cell experiments have shown that OTA can reduce the contents of glucocorticoids in human adrenocortical carcinoma cells [48]. Mercado et al. [49] found that glucocorticoid receptor can reduce the expression of the IL-1β gene in mouse skin. A study in humans showed that reduced IL-1β concentrations can increase epidermal keratinocyte claudin-3 protein [50]. However, studies on Mozambique tilapia (*Oreochromis mossambicus*) have shown that the gene expression levels of the glucocorticoid receptor in the PI are lower than those in the MI and DI [51]. Therefore, OTA decreased claudin-3c expression only in the MI and DI (but not in the PI) of juvenile grass carp, which may be related to the lower glucocorticoid receptor levels in the PI, but the mechanism of action needs further research. Thirdly, OTA down-regulated ZO-2b gene expression only in the PI, which might be connected with TGF-β2 and PKC. A study has shown that OTA can promote the expression of TGF-β2 mRNA in male rats [52], and TGF-β2 increased the phosphorylation level of PKC in human retinal pigment epithelial cells [53]; PKC is necessary for tightly regulated ZO-2b phosphorylation [54]. In this study, we speculated that the fact that OTA does not change the expression of the ZO-2b gene (in the MI and DI) may be related to TGF-β2. However, this speculation needs further verification.

### 3.4. The Maximum Controlled Dose of OTA for Juvenile Grass Carp

In this experiment, we used broken-line regression analysis to evaluate the maximum controlled dose of OTA (25–125 g) in juvenile grass carp according to different indicators (Table 3). Based on the PWG and specific growth rate (SGR), the maximum controlled doses of OTA were 803.53 and 874.33 µg/kg of diet, respectively. As seen in the ROS contents of the PI, MI and DI, the maximum controlled doses were 476.78, 609.42 and 390.67 µg/kg of diet, respectively. The maximum controlled levels determined according to the MDA contents of the PI, MI and DI were 404.79, 469.64 and 384.53 µg/kg of diet, respectively. These data suggest that the maximum controlled doses of OTA for guaranteeing fish intestinal structure were lower than those for fish growth. Our finding is similar to that of Wang et al. [31], who evaluated the maximum controlled doses of ZEA for fish growth and intestinal structure in juvenile grass carp. In addition, our laboratory has found similar results in other studies on toxic and harmful substances in feed. In the growing grass carp, the maximum tolerable levels of gossypol [33,55] and erucic acid [56,57] for the structural integrity of the fish intestine are lower than the levels for fish growth.

## 4. Conclusions

**Figure 8:**
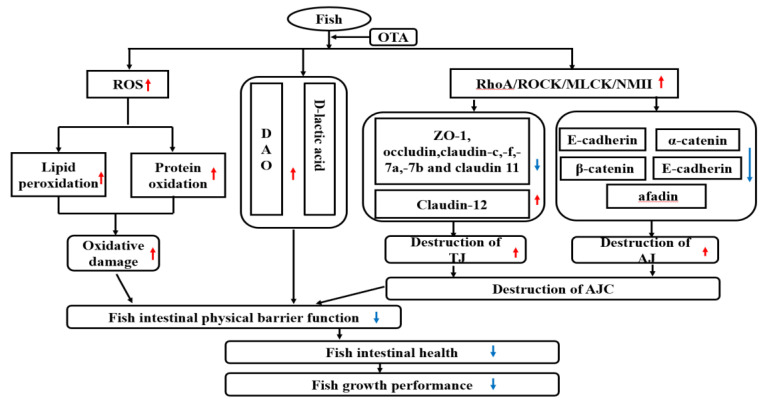
The potential action pathways of dietary OTA disruption of intestinal physical barrier function in fish. Blue arrows: down-regulation; red arrows: up-regulation.

In summary, as shown in Figure 8, this is the first paper to comprehensively explain that a certain dose of OTA can inhibit growth and result in the destruction of the intestinal structural integrity of fish, which may help to better elucidate the toxic effects of OTA in fish. Some of the many new findings in this research are as follows: OTA (1) depressed the feed efficiency, percentage weight gain and specific growth rate; (2) accumulated in the intestine; (3) caused oxidative damage and increased intestinal permeability; and (4) induced the RhoA/ROCK signaling pathway, destroying intestinal apical junctional complexes. Notably, OTA intervention did not affect the gene expression of claudin-3c (PI), claudin-b and ZO-2b (in the MI and DI) in the fish intestine.

## 5. Materials and Methods

### 5.1. Diets

The basic composition of the diet is listed in Table 4. Fish meal, casein, rice flour and gelatin provide the main protein. Dietary lipids are mainly provided by soybean oil and fish oil. Seven different OTA (purity > 99%, purchased from Pribolab Pte, Ltd., Singapore) concentrations were used: 0 (control group), 400, 800, 1200, 1600, 2000 and 2400 µg/kg of diet. The experiment was performed according to the addition method of Sahoo et al. [58] and our improvement. We dissolved 1 mg of OTA in 1 mL of ethanol, re-dissolved the mixture in 10 mL of soybean oil (0.05 mg of OTA per 0.5 mL of soybean oil), placed the mixture in a fume hood for 2 h to evaporate the ethanol, and then, according to the additive amount for each group, mixed the feed sample. The actual OTA concentrations in the diet were determined by high-performance liquid chromatography (HPLC) to be 0, 406, 795, 1209, 1612, 2003 and 2406 µg of OTA/kg of diet. Finally, we preserved the diets at −20 °C until needed [10].

### 5.2. Feeding Trial and Sample Collection

The breeding and management of this study were approved by the University of Sichuan Agricultural Animal Care Advisory Committee, Sichuan, China, under permit No. LX-S20176966. Juvenile grass carp were obtained from fisheries (Sichuan, China). We domesticated grass carp for 4 weeks before the formal test, referring to the method of Pan et al. [61]. After that, 1260 fish (average weight, 25.73 (SD, 0.04) g) were, at random, allocated to 21 experimental cages (1.5 L × 0.8 W × 1.5 H), which eventually led to three cages in each treatment and 60 fish in each cage. Fish were fed the respective diets four times per day over a period of 60 consecutive days. After 30 minutes of feeding, the surplus feed was collected, dried and weighed to calculate the feed intake (FI) according to the method of Dong et al. [62]. During the period of the feed trial, the dissolved oxygen was at least 6.0 mg/L, and according to the methods of measurement mentioned by Li et al. [63], the pH and water temperature were 7.4 (SD, 0.4) and 22.5 (SD, 2.5) °C, respectively. The growth trial was conducted under natural light and dark cycles, similar to Liu et al. [38]. At the end of the experiment, the fish in each cage were weighed, and 45 fish of close weights were selected and anaesthetized in a benzocaine bath before sacrifice. Then, the fish intestine was divided into the proximal intestine (PI), mid intestine (MI) and distal intestine (DI), quickly frozen in liquid nitrogen and stored at −80 °C for analysis as mentioned by Zeng et al. [64]. To conduct the intestinal histological examination, we collected section samples from each treatment and fixed them in a 4% paraformaldehyde solution according to Nagel et al. [65]. A blood sample for each treatment group was centrifuged at 4000× *g* for 15 minutes, and then, the serum was taken and stored at −20 °C [66] for the subsequent detection of serum DAO and D-lactic acid.

### 5.3. Biochemical Analysis

We used 10 vol. (*w*/*v*) of ice-cold physiological saline to homogenize all the samples, used a centrifuge at the conditions of 4 °C and 6000× *g* to centrifuge them for 20 minutes, and collected the supernatants [67] for the subsequent detection of ROS (reactive oxygen species), PC (protein carbonyl) and MDA (malondialdehyde). The ROS Assay Kit, purchased from Clover Technology Group (Beijing, China), was used to detect the production of ROS according to Soraya et al. [68]. The tests for the MDA and PC contents were conducted using the MDA and PC Assay Kits (Nanjing Jiancheng Bioengineering Institute, Nanjing, China), respectively, according to the method of Yu et al. [69]. Using an ELISA kit purchased from Pribolab Pte, Ltd. (Tanjung Bago, Singapore), residues of OTA in the intestine were detected [70].

### 5.4. Histological Examination

The sampling of three fish intestines from each treatment was performed for histomorphological evaluation according to Morán et al. [71]. Briefly, 4 µm-thickness intestinal tissues were stained following the protocols for hematoxylin and eosin (H&E), and then, the slides were observed under a light microscope (Olympus, DP72) equipped with a camera (Nikon TS100).

### 5.5. RT-PCR Analysis

Total RNA (ribonucleic acid) was extracted using the RNAiso Plus kit (Takara, Dalian, China), and its concentration, integrity and quality were detected by spectrophotometry [72]. Then, according to the instructions for the PrimeScript RT Reagent Kit (Takara, Dalian, China), RNA was reverse transcribed to synthesize cDNA. Table 5 presents the sequences of the primers used. After that, fluorescent quantitative PCR amplification was conducted using SYBR (Takara, Dalian, China), and after the amplification was completed, a dissolution curve analysis was performed to determine the amplification results, ensuring that the amplification efficiency was close to 100%. According to the pre-experimental results, β-actin was chosen as the internal reference gene. The relative expression levels of genes were measured with the 2^−∆∆CT^ method [73].

### 5.6. Western Blot Analysis

The procedure of Western blotting (WB) was performed to measure the GTP-RhoA and RhoA proteins in the fish intestines according to Niu et al. [74]. In short, after extraction, the concentrations of three intestinal segment proteins were examined using an assay kit (Bio-Rad, Hercules, CA, USA). The protein samples were separated by SDS-PAGE (the target protein loading was 40 µg of protein per lane) and transferred to a PVDF (0.45 µm) membrane for Western analysis. Then, we blocked the PVDF membrane at room temperature (RT) for 90 minutes, and then incubated it with RhoA antibody (primary antibody) at 4 °C overnight. After the primary antibody incubation, the membrane was washed 3 times for 5 minutes each time, and then, the washed membrane was incubated with the secondary antibody (goat anti-rabbit secondary antibody) with horseradish peroxidase (Shanghai Biyuntian Biotechnology Co., Ltd.) for 90 minutes. The GTP-RhoA analysis employed the total RhoA as the control protein. After washing, the immune complexes were observed using an ECL kit (Beyotime Biotechnology Inc., Jiangsu, China). The signal strength was analyzed using the NIH Image 1.63 software.

### 5.7. Statistical Analysis

The test data are presented as mean ± standard deviation (SD). Using the SPSS 18.0 software (SPSS Inc., Chicago, Illinois, United States), Duncan’s multi-range test was used to assess significant differences between treatments by one-way analysis of variance (ANOVA). According to the means and standard deviations of the intestinal structure-related parameters, the minimum effect size was 0.56 on the basis of Searcy Bernal’s work [75]. The effect size was 0.56, the significance level of differences was 0.05, and each treatment was repeated 6 times. Referring to the method of Gray et al. [76], the statistical power calculated using the R pwr package was 0.70. Using the SGR, PWG and intestinal antioxidant indicators, the maximum controlled levels of OTA were estimated by a broken-line model [32]. In Table 6 are shown the growth indicators calculated according to Li et al. [77], and the ILI and ISI were calculated according to Jiang et al. [78].

## Figures and Tables

**Table 1:** Growth performance, intestinal growth and ochratoxin A (OTA) residue (µg/kg of tissue) of juvenile grass carp (*Ctenopharyngodon idella*) fed diets with graded levels of OTA over a 60-day period.

Growth Indicators	Dietary OTA Levels (µg/kg Diet)						
	0.0	406	795	1209	1612	2003	2406
IBW ^1^	25.73 ± 0.07 ^a^	25.74 ± 0.03 ^a^	25.70 ± 0.08 ^a^	25.79 ± 0.07 ^a^	25.76 ± 0.09 ^a^	25.68 ± 0.04 ^a^	25.75 ± 0.13 ^a^
FBW ^1^	125.22 ± 2.14 ^e^	125.00 ± 1.20 ^e^	124.67 ± 0.88 ^e^	117.22 ± 3.53 ^d^	108.78 ± 3.40 ^c^	99.89 ± 1.26 ^b^	92.44 ± 2.14 ^a^
PWG ^1^	386.59 ± 7.10 ^e^	385.63 ± 4.64 ^e^	385.07 ± 3.23 ^e^	354.48 ± 14.25 ^d^	322.34 ± 13.75 ^c^	289.01 ± 5.48 ^b^	259.05 ± 10.10 ^a^
SGR ^1^	2.637 ± 0.024 ^e^	2.634 ± 0.015 ^e^	2.632 ± 0.011 ^e^	2.523 ± 0.052 ^d^	2.406 ± 0.053 ^c^	2.264 ± 0.023 ^b^	2.130 ± 0.0472 ^a^
FI ^1^	122.19 ± 0.12 ^e^	122.15 ± 0.14 ^e^	122.08 ± 0.17 ^e^	115.21 ± 0.11 ^d^	109.97 ± 0.18 ^c^	101.67 ± 0.18 ^b^	95.56 ± 0.20 ^a^
FE ^1^	0.81 ± 0.02 ^c^	0.81 ± 0.01 ^c^	0.81 ± 0.01 ^c^	0.79 ± 0.05 ^c^	0.75 ± 0.05 ^b^	0.73 ± 0.02 ^b^	0.70 ± 0.05 ^a^
IL ^2^	33.13 ± 2.47 ^c^	32.39 ± 1.20 ^c^	31.72 ± 1.03 ^c^	28.02 ± 0.71 ^b^	26.78 ± 0.47 ^b^	23.88 ± 0.57 ^a^	23.62 ± 0.18 ^a^
ILI ^2^	167.59 ± 5.50 ^c^	162.28 ± 8.67 ^c^	161.96 ± 0.49 ^c^	152.39 ± 5.34 ^bc^	143.32 ± 6.48 ^b^	132.38 ± 3.29 ^a^	124.16 ± 2.18 ^a^
IW ^2^	3.30 ± 0.05 ^d^	3.21 ± 0.06 ^d^	3.16 ± 0.08 ^d^	2.84 ± 0.14 ^c^	2.49 ± 0.12 ^b^	2.17 ± 0.07 ^a^	2.00 ± 0.11 ^a^
ISI ^2^	2.69 ± 0.07 ^c^	2.60 ± 0.05 ^bc^	2.60 ± 0.15 ^bc^	2.46 ± 0.08 ^b^	2.21 ± 0.10 ^a^	2.21 ± 0.12 ^a^	2.15 ± 0.15 ^a^
OTA Content (μg/kg tissue)							
PI ^3^	n.d ^4^	n.d ^4^	n.d ^4^	8.02 ± 0.20 ^a^	16.68 ± 1.26 ^b^	28.68 ± 2.85 ^c^	39.24 ± 3.55 ^d^
MI ^3^	n.d ^4^	n.d ^4^	n.d ^4^	15.14 ± 1.09 ^a^	26.13 ± 1.54 ^b^	32.67 ± 2.93 ^c^	41.19 ± 1.73 ^d^
DI ^3^	n.d ^4^	n.d ^4^	9.33 ± 0.77 ^a^	21.22 ± 2.03 ^b^	28.61 ± 2.74 ^c^	34.17 ± 3.28 ^d^	49.56 ± 2.75 ^e^

^1^ Values are means ± SD for three replicate groups, with 60 fish in each group, and values in the same column with different superscripted small letters mean significant differences (*p* < 0.05). IBW, initial body weight (g/fish); FBW, final body weight (g/fish); PWG, percent weight gain (%); SGR, specific growth rate (%/day); FI, feed intake (g/fish); FE, feed efficiency. ^2^ Values are means ± SD (*n* = 42), and values in the same column with different superscripted same letters mean significant differences (*p* < 0.05). IL, intestinal length (cm); ILI, intestinal length index; IW, intestinal weight (g/fish); ISI, intestinal somatic index. ^3^ Values are means ± SD (*n* = 6), and values in the same column with different superscripted same letters mean significant differences (*p* < 0.05). ^4^ n.d: not detected.

**Table 2:** Oxidation-related parameters in the PI, MI and DI of juvenile grass carp (*Ctenopharyngodon idella*) fed diets containing graded levels of OTA over a 60-day period.

Item	Dietary OTA Levels (µg/kg Diet)						
	0	406	795	1209	1612	2003	2406
PI							
ROS	100.00 ± 7.74 ^a^	102.41 ± 3.42 ^a^	102.61 ± 1.84 ^a^	115.69 ± 3.54 ^b^	120.34 ± 6.68 ^bc^	124.56 ± 3.28 ^c^	125.32 ± 7.80 ^c^
MDA	17.82 ± 1.52 ^a^	17.99 ± 0.90 ^a^	18.08 ± 0.27 ^a^	23.70 ± 2.04 ^b^	24.86 ± 1.95 ^bc^	25.32 ± 2.08 ^bc^	26.74 ± 1.82 ^c^
PC	2.94 ± 0.28 ^a^	3.00 ± 0.21 ^a^	3.04 ± 0.19 ^a^	4.74 ± 0.26 ^b^	5.10 ± 0.40 ^b^	7.30 ± 0.70 ^c^	8.89 ± 0.57 ^d^
MI							
ROS	100.00 ± 9.76 ^a^	103.70 ± 2.81 ^a^	104.43 ± 2.88 ^a^	110.95 ± 7.34 ^b^	114.62 ± 4.20 ^bc^	116.01 ± 4.06 ^bc^	120.90 ± 2.46 ^c^
MDA	16.53 ± 1.06 ^a^	16.66 ± 0.91 ^a^	16.85 ± 0.71 ^a^	22.94 ± 1.40 ^b^	23.60 ± 0.80 ^b^	25.76 ± 1.89 ^c^	26.65 ± 1.11 ^c^
PC	2.99 ± 0.18 ^a^	3.03 ± 0.16 ^a^	3.09 ± 0.19 ^a^	3.92 ± 0.36 ^b^	4.34 ± 0.25 ^b^	6.88 ± 0.37 ^c^	7.00 ± 0.68 ^c^
DI							
ROS	100.00 ± 3.45 ^a^	104.83 ± 2.60 ^a^	105.13 ± 3.38 ^a^	112.30 ± 7.24 ^b^	114.31 ± 6.05 ^b^	115.11 ± 7.03 ^b^	116.84 ± 6.75 ^b^
MDA	17.21 ± 0.43 ^a^	17.61 ± 1.58 ^a^	17.89 ± 0.74 ^a^	23.94 ± 0.90 ^b^	25.29 ± 1.61 ^bc^	26.77 ± 2.11 ^c^	26.80 ± 1.33 ^c^
PC	2.95 ± 0.24 ^a^	2.99 ± 0.10 ^a^	3.06 ± 0.24 ^a^	5.08 ± 0.44 ^b^	5.19 ± 0.47 ^b^	5.51 ± 0.28 ^b^	6.53 ± 0.61 ^c^

Values are means ± SD (*n* = 6), and values in the same column with different superscripted small letters mean significant differences (*p* < 0.05). ROS, reactive oxygen species (% DCF fluorescence); MDA, malondialdehyde (nmol/g of tissue); PC, protein carbonyl (nmol/mg of protein).

**Table 3:** The maximum allowable doses of OTA based on different indexes (µg/kg).

Regression Equation	Regression	*p*	The Maximum Allowable Doses of OTA (µg/kg)
Growth performance			
Y_PWG_ = −0.0794x + 449.56 Y_min_ = 386.53	0.9996	<0.01	803.53 µg/kg
Y_SGR_ = −0.0003x + 2.8965 Y_min_ = 2.63	0.9975	<0.01	874.33 µg/kg
PI			
Y_ROS_ = 0.014x + 94.995 Y_min_ = 101.67	0.8516	<0.05	476.78 µg/kg
Y_MDA_ = 0.0048x + 16.022 Y_min_ = 17.96	0.8043	<0.05	404.79 µg/kg
MI			
Y_ROS_ = 0.0103x + 96.430 Y_min_ = 102.71	0.9370	<0.01	609.42 µg/kg
Y_MDA_ = 0.0057x + 14.00 Y_min_ = 16.68	0.8521	<0.05	469.49 µg/kg
DI			
Y_ROS_ = 0.0075x + 100.39 Y_min_ = 103.32	0.7946	<0.05	390.67 µg/kg
Y_MDA_ = 0.0053x + 15.531 Y_min_ = 17.57	0.7812	<0.05	384.53 µg/kg

**Table 4:** Composition and nutrient contents of basal diet.

Ingredients	%	Nutrient Content	%
Fish meal	7.30	Crude protein ^d^	32.22
Casein	23.50	Crude lipid ^d^	4.86
Gelatin	6.83	n-3 ^e^	1.04
α-starch	24.00	n-6 ^e^	0.96
Rice flour	13.764	Available phosphorus ^f^	0.84
Fish oil	1.44		
Soybean oil	1.81		
Cellulose	5.00		
Ca(H_2_PO_4_)_2_	3.13		
Vitamin premix ^a^	1.00		
Mineral premix ^b^	2.00		
Choline chloride (50%)	1.00		
Ethoxyquin (30%)	0.050		
Trp (99.2%)	0.031		
Thr (98.5%)	0.145		
OTA premix ^c^	9.00		

^a^ Per kilogram of vitamin premix (g/kg): cholecalciferol (500,000 IU/g), 0.320; retinyl acetate (1000,000 IU/g), 0.400; menadione (96%), 0.198; DL-a-tocopherol acetate (50%), 40.000; D-biotin (2%), 0.750; cyanocobalamin (1%), 0.940; thiamine nitrate (98%), 0.133; folic acid (95%), 0.379; meso-inositol (97%), 22.068; ascorbyl acetate (95%) and calcium-D-pantothenate (90%), 2.778 and 4.737; niacin (99%), 2.576; pyridoxine hydrochloride (98%), 0.115; riboflavin (80%), 0.775. All ingredients were diluted with maize starch to 1 kg. ^b^ Per kilogram of mineral premix (g/kg): CuSO_4_.5H_2_O (25.0% Cu), 0.600; MnSO_4_.H_2_O (31.8% Mn), 3.098; FeSO_4_.H_2_O (30.0% Fe), 15.000; MgSO_4_.H_2_O (15.0% Mg), 237.840; Na_2_SeO_3_ (44.7% Se), 0.132; CaI_2_ (3.2% I), 1.560; ZnSO_4_.H_2_O (34.5% Zn), 7.681. All ingredients were diluted with maize starch to 1 kg. ^c^ OTA premix: premix was added to obtain graded levels of ochratoxin A. ^d^ Crude protein and crude lipid contents: measured values. ^e^ n-3 and n-6 contents were calculated with reference to Zeng et al. [59]. ^f^ Available phosphorus content was calculated with reference to Liang et al. [60].

**Table 5:** Real-time PCR primer sequences ^1^.

Target Gene	Primer Sequence, Forward (5′→3′)	Primer Sequence, Reverse (5′→3′)	Temperature (°C)	Accession Number
ZO-1	CGGTGTCTTCGTAGTCGG	CAGTTGGTTTGGGTTTCAG	59.4	KJ000055
Occludin	TATCTGTATCACTACTGCGTCG	CATTCACCCAATCCTCCA	59.4	KF193855
ZO-2b	TACAGCGGGACTCTAAAATGG	TCACACGGTCGTTCTCAAAG	60.3	KM112095
Claudin-b	GAGGGAATCTGGATGAGC	ATGGCAATGATGGTGAGA	57.0	KF193860
JAM-A	ACTGTGAGGTGCTTGGAA	CTGTTGTGACTGAAGAAGGA	61.4	KY780630
Claudin-c	GAGGGAATCTGGATGAGC	CTGTTATGAAAGCGGCAC	59.4	KF193859
RhoA	GCAGGACAAGAGGACTATG	GTGTTCATCATTCCGTAGGT	63.3	MN661351
Claudin-f	GCTGGAGTTGCCTGTCTTATTC	ACCAATCTCCCTCTTTTGTGTC	57.1	KM112097
Claudin-3c	ATCACTCGGGACTTCTA	CAGCAAACCCAATGTAG	57.0	KF193858
ROCK	AGTCCAAGTCTGCTGCTA	CCTCTCCTTCTGCTTCATC	63.3	KY780630
Claudin-7a	ACTTACCAGGGACTGTGGATGT	CACTATCATCAAAGCACGGGT	59.3	KT625604
Claudin-7b	CTAACTGTGGTGGTGATGAC	AACAATGCTACAAAGGGCTG	59.3	KT445866
Claudin-11	TCTCAACTGCTCTGTATCACTGC	TTTCTGGTTCACTTCCGAGG	62.3	KT445867
Nectin	GCCAGTGACCAAGATGAC	ACAGTGCCATTCGGATTG	61.4	MN661350
Claudin-12	CCCTGAAGTGCCCACAA	GCGTATGTCACGGGAGAA	55.4	KF998571
Claudin-15a	TGCTTTATTTCTTGGCTTTC	CTCGTACAGGGTTGAGGTG	59.0	KF193857
Claudin-15b	AGTGTTCTAAGATAGGAGGGGAG	AGCCCTTCTCCGATTTCAT	62.3	KT757304
Afadin	CCTGTGCTCACACTACTG	GTCGTTGCCTGGACTATG	61.4	MN661352
E-cadherin	GACTGTAACGCTGAAGAGA	CTGTGGAGAGGAGATGTTC	61.4	MN661354
α-catenin	GCAATCTTCTCTCCTTTATCC	ACTTGTGAACTCCAGCAAT	61.4	HQ338751
β-catenin	GTCTGCTTGCCATCTTCA	CAGGTTGTGTAGAGTCGTAA	64.5	MN661349
MLCK	GAAGGTCAGGGCATCTCA	GGGTCGGGCTTATCTACT	53.0	KM279719
NMII	AGCCAACTCGTCAATGTC	CCTTGGAATACTTCTCTGTCT	61.4	MN661353
β-actin	GGCTGTGCTGTCCCTGTA	GGGCATAACCCTCGTAGAT	61.4	M25013

^1^ ZO, zonula occludins; MLCK, myosin light chain kinase; JAM-A, junctional adhesion molecule-A; NMII, non-muscle myosin II; ROCK, Rho associated protein kinase; RhoA, a small Rho GTPase protein.

**Table 6:** Computational formulas.

Growth Indicators	Formulas
PWG	100 × [FBW (g/fish) − IBW (g/fish)]/IBW (g/fish)
SGR	100 × [In (mean final weight − In (mean initial weight)]/days
FE	[FBW (g/fish) − IBW (g/fish)]/FI (g/fish)
ISI	100 × [wet intestine weight (g)/wet body weight (g)]
ILI	100 × [intestine length (cm)/total body length (cm)]

## Supplementary Materials

The following are available online at https://www.mdpi.com/2072-6651/13/1/11/s1. Table S1: Correlation analysis of parameters in the intestine of juvenile grass carp.
