# Supplementary material for: Effects of Dietary Ochratoxin A on Growth Performance and Intestinal Apical Junctional Complex of Juvenile Grass Carp (Ctenopharyngodon idella)
Source: Toxins (Basel). 2020 Dec 24;13(1):11. doi: 10.3390/toxins13010011 (PMC7823973; doi:10.3390/toxins13010011)
Supplement: Supplementary file 1 [file toxins-13-00011-s001.pdf]

Supplementary Materials: Effects of Dietary Ochratoxin A on Growth Performance and Intestinal Apical Junctional Complex of Juvenile Grass Carp (Ctenopharyngodon idella)

Xin Liu, Pei Wu, Wei-Dan Jiang, Yang Liu, Jun Jiang, Sheng-Yao Kuang, Ling Tang, Xiao-Qiu Zhou and Lin Feng

Table S1. Correlation analysis of parameters in the intestine of juvenile grass carp.

Independent Parameters	Dependent Parameters	PI		MI		DI	
		Correlation Coefficients	P	Correlation Coefficients	P	Correlation Coefficients	P
RhoA	ZO [1]-1	−0.913	<0.01	−0.982	<0.01	−0.965	<0.01
	ZO [1]-2b	−0.982	<0.01	-	-	-	-
	occludin	−0.970	<0.01	−0.968	<0.01	−0.972	<0.01
	claudin-b	-	-	-	-	-	-
	claudin-c	−0.922	<0.01	−0.971	<0.01	−0.993	<0.01
	claudin-f	−0.931	<0.01	−0.962	<0.01	−0.984	<0.01
	claudin-3c	-	-	−0.961	<0.01	−0.995	<0.01
	claudin-7a	−0.987	<0.01	−0.971	<0.01	−0.955	<0.01
	claudin-7b	−0.896	<0.01	−0.987	<0.01	−0.959	<0.01
	claudin-11	−0.981	<0.01	−0.973	<0.01	−0.988	<0.01
	claudin-12	0.906	<0.01	0.983	<0.01	0.932	<0.01
	claudin-15a	−0.909	<0.01	−0.974	<0.01	−0.993	<0.01
	claudin-15b	0.916	<0.01	−0.976	<0.01	−0.995	<0.01
	JAM [2]-A	−0.983	<0.01	−0.956	<0.01	−0.980	<0.01
	E-cadherin	−0.968	<0.01	−0.970	<0.01	−0.941	<0.01
	α-catenin	−0.953	<0.01	−0.942	<0.01	−0.993	<0.01
	β-catenin	−0.921	<0.01	−0.953	<0.01	−0.973	<0.01
	nectin	−0.990	<0.01	−0.975	<0.01	0.990	<0.01
	afadin	−0.968	<0.01	−0.993	<0.01	−0.988	<0.01
RhoA protein level	ZO [1]-1	−0.938	<0.01	−0.969	<0.01	−0.776	<0.05
	ZO [1]-2b	−0.884	<0.01	-	-	-	-
	occludin	−0.875	<0.01	−0.965	<0.01	−0.840	<0.05
	claudin-b	-	-	-	-	-	-
	claudin-c	−0.919	<0.01	−0.967	<0.01	−0.878	<0.01
	claudin-f	−0.899	<0.01	−0.951	<0.01	−0.921	<0.01
	claudin-3c	-	-	−0.975	<0.01	−0.880	<0.01
	claudin-7a	−0.876	<0.01	−0.965	<0.01	−0.955	<0.01
	claudin-7b	−0.965	<0.01	−0.987	<0.01	−0.833	<0.05
	claudin-11	−0.867	<0.05	−0.938	<0.01	−0.896	<0.01
	claudin-12	0.901	<0.01	0.963	<0.01	0.957	<0.01
	claudin-15a	−0.983	<0.01	−0.993	<0.01	−0.922	<0.01
	claudin-15b	−0.924	<0.01	−0.909	<0.01	−0.907	<0.01
	JAM [2]-A	−0.846	<0.05	−0.964	<0.01	−0.830	<0.05
	E-cadherin	−0.936	<0.01	−0.948	<0.01	−0.773	<0.05
	α-catenin	−0.925	<0.01	−0.963	<0.01	−0.902	<0.01
	β-catenin	−0.923	<0.01	−0.966	<0.01	−0.967	<0.01
	nectin	−0.872	<0.05	−0.970	<0.01	−0.879	<0.01
	afadin	−0.936	<0.01	−0.968	<0.01	−0.903	<0.01
ROCK	ZO [1]-1	−0.945	<0.01	−0.946	<0.01	−0.987	<0.01
	ZO [1]-2b	−0.990	<0.01	-	-	-	-
	occludin	−0.993	<0.01	−0.933	<0.01	−0.980	<0.01
	claudin-b	-	-	-	-	-	-

	claudin-c	−0.953	<0.01	−0.933	<0.01	−0.988	<0.01
	claudin-f	−0.961	<0.01	−0.909	<0.01	−0.933	<0.01
	claudin-3c	-	-	−0.928	<0.01	−0.968	<0.01
	claudin-7a	−0.980	<0.01	−0.922	<0.01	−0.892	<0.01
	claudin-7b	−0.934	<0.01	−0.962	<0.01	−0.968	<0.01
	claudin-11	−0.968	<0.01	−0.927	<0.01	−0.950	<0.01
	claudin-12	0.931	<0.01	0.943	<0.01	0.880	<0.01
	claudin-15a	−0.933	<0.01	−0.939	<0.01	−0.947	<0.01
	claudin-15b	−0.934	<0.01	−0.939	<0.01	−0.968	<0.01
	JAM [2]-A	−0.989	<0.01	−0.928	<0.01	−0.989	<0.01
	E-cadherin	−0.977	<0.01	−0.925	<0.01	−0.954	<0.01
	α-catenin	−0.981	<0.01	−0.927	<0.01	−0.974	<0.01
	β-catenin	−0.942	<0.01	−0.920	<0.01	−0.914	<0.01
	nectin	−0.995	<0.01	−0.946	<0.01	−0.962	<0.01
	afadin	−0.977	<0.01	−0.961	<0.01	−0.949	<0.01
	ZO [1]-1	−0.981	<0.01	−0.995	<0.01	−0.972	<0.01
	ZO [1]-2b	−0.934	<0.01	-	-	-	-
	occludin	−0.932	<0.01	−0.948	<0.01	−0.969	<0.01
	claudin-b	-	-	-	-	-	-
	claudin-c	−0.963	<0.01	−0.941	<0.01	−0.990	<0.01
	claudin-f	−0.953	<0.01	−0.944	<0.01	−0.981	<0.01
	claudin-3c	-	-	−0.934	<0.01	−0.998	<0.01
	claudin-7a	−0.915	<0.01	−0.952	<0.01	−0.947	<0.01
	claudin-7b	−0.996	<0.01	−0.968	<0.01	−0.951	<0.01
MLCK	claudin-11	−0.903	<0.01	−0.969	<0.01	−0.985	<0.01
	claudin-12	0.954	<0.01	0.998	<0.01	0.918	<0.01
	claudin-15a	−0.936	<0.01	−0.968	<0.01	−0.990	<0.01
	claudin-15b	−0.966	<0.01	−0.979	<0.01	−0.993	<0.01
	JAM [2]-A	−0.899	<0.01	−0.955	<0.01	0.984	<0.01
	α-catenin	−0.970	<0.01	−0.887	<0.01	−0.987	<0.01
	β-catenin	−0.961	<0.01	−0.960	<0.01	−0.948	<0.01
	E-cadherin	−0.929	<0.01	−0.974	<0.01	−0.939	<0.01
	afadin	−0.929	<0.01	−0.982	<0.01	−0.976	<0.01
	nectin	−0.916	<0.01	−0.981	<0.01	−0.983	<0.01
	ZO [1]-1	−0.966	<0.01	−0.981	<0.01	−0.976	<0.01
	ZO [1]-2b	−0.953	<0.01	-	-	-	-
	occludin	−0.937	<0.01	−0.924	<0.01	−0.974	<0.01
	claudin-b	-	-	-	-	-	-
	claudin-c	−0.951	<0.01	−0.923	<0.01	−0.988	<0.01
	claudin-f	−0.944	<0.01	−0.930	<0.01	−0.929	<0.01
	claudin-3c	-	-	−0.906	<0.01	−0.962	<0.01
	claudin-7a	−0.952	<0.01	−0.938	<0.01	−0.894	<0.01
	claudin-7b	−0.976	<0.01	−0.950	<0.01	0.965	<0.01
NMII	claudin-11	−0.946	<0.01	−0.956	<0.01	0.945	<0.01
	claudin-12	0.948	<0.01	0.983	<0.01	0.887	<0.01
	claudin-15a	−0.927	<0.01	−0.946	<0.01	−0.946	<0.01
	claudin-15b	−0.965	<0.01	−0.988	<0.01	−0.969	<0.01
	JAM [2]-A	−0.922	<0.01	−0.946	<0.01	−0.981	<0.01
	E-cadherin	−0.955	<0.01	−0.978	<0.01	−0.946	<0.01
	α-catenin	−0.968	<0.01	−0.866	<0.01	−0.974	<0.01
	β-catenin	−0.958	<0.01	−0.946	<0.01	−0.929	<0.01
	nectin	−0.940	<0.01	−0.970	<0.01	−0.962	<0.01
	afadin	−0.955	<0.01	−0.983	<0.01	−0.959	<0.01

[1] ZO, zonula occluden. [2] JAM, junctional adhesion molecule.
